# Tunable Thermoelastic Anisotropy in Hybrid Bragg Stacks with Extreme Polymer Confinement

**DOI:** 10.1002/anie.201911546

**Published:** 2019-12-04

**Authors:** Zuyuan Wang, Konrad Rolle, Theresa Schilling, Patrick Hummel, Alexandra Philipp, Bernd A. F. Kopera, Anna M. Lechner, Markus Retsch, Josef Breu, George Fytas

**Affiliations:** ^1^ Max Planck Institute for Polymer Research Ackermannweg 10 55128 Mainz Germany; ^2^ Bavarian Polymer Institute University of Bayreuth Universitätsstraße 30 95447 Bayreuth Germany; ^3^ Department of Chemistry University of Bayreuth Universitätsstraße 30 95447 Bayreuth Germany; ^4^ Institute of Electronic Structure and Laser, F.O.R.T.H 70013 Heraklion Greece

**Keywords:** anisotropy, Brillouin light scattering, mechanical properties, organic–inorganic hybrid composites, thermal conductivity

## Abstract

Controlling thermomechanical anisotropy is important for emerging heat management applications such as thermal interface and electronic packaging materials. Whereas many studies report on thermal transport in anisotropic nanocomposite materials, a fundamental understanding of the interplay between mechanical and thermal properties is missing, due to the lack of measurements of direction‐dependent mechanical properties. In this work, exceptionally coherent and transparent hybrid Bragg stacks made of strictly alternating mica‐type nanosheets (synthetic hectorite) and polymer layers (polyvinylpyrrolidone) were fabricated at large scale. Distinct from ordinary nanocomposites, these stacks display long‐range periodicity, which is tunable down to angstrom precision. A large thermal transport anisotropy (up to 38) is consequently observed, with the high in‐plane thermal conductivity (up to 5.7 W m^−1^ K^−1^) exhibiting an effective medium behavior. The unique hybrid material combined with advanced characterization techniques allows correlating the full elastic tensors to the direction‐dependent thermal conductivities. We, therefore, provide a first analysis on how the direction‐dependent Young's and shear moduli influence the flow of heat.

## Graphical abstract

**Hybrid Bragg stacks**, consisting of alternating hectorite and polyvinylpyrrolidone layers, are fabricated with Ångstrom precision. By controlling the sample composition, tunable mechanical and thermal anisotropy ratios, up to 7 and 38 respectively, are achieved.

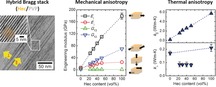

## Introduction

Heat management is crucial in many applications important for fueling the growth of our technology‐driven society. It needs to address not only very small length scales to dissipate the heat produced, for example, by electronic circuits, but also very large length scales to realize air conditioning, for instance, for commercial buildings. The ubiquity of heat makes it obvious that heat management is a key technology to realize international long‐term goals regarding global warming. Controlling the elusive flow of heat is a complex challenge across multiple materials, length scales, and ultimately devices. This results in stringent requirements for directional control over the heat flux based on advanced material design. Whereas heat transport represents an effective, far‐field phenomenon, it is decisively governed by the material structure^1^ and chemistry^2^ on the microscale. Extreme phenomena of both heat dissipation and thermal insulation have been demonstrated in nanostructured and hybrid materials. For heat dissipation, surprisingly high thermal conductivities have been reported for one‐dimensional (1D) fibers comprising synthetic^3^ and natural polymers.^4^ For thermal insulation, unusually low thermal conductivities have been shown for (disordered) stacks of two‐dimensional (2D) materials.^5^ Extremely efficient anisotropic thermal insulation materials have been demonstrated with various mixtures of polymers and nanoparticles or 2D materials.^6^

The combination of inherently different materials, such as soft and hard matter, is attractive, as new properties, deviating from those based on the simple linear interpolation, could emerge. This is often accompanied by improved processability, which is aided by the complementary properties of the constituent components. For instance, the soft component can serve as a binder to enable fabrication of large‐area, thin films of an otherwise brittle, hard component. On the contrary, the expected effective material properties, such as mechanical reinforcement, optical transparency, and electrical or thermal conductivity, have been often found inferior to the high expectations. The reason for such shortcomings is that the nanocomposite structure, particularly the soft‐hard interface, is poorly controlled. Furthermore, although many characterization techniques, such as tensile testing, indentation, and abrasion tests, are capable of assessing engineering properties, they are unsuitable for directly identifying and quantifying anisotropies or microscopic contributions to the effective properties.

Nevertheless, hybrid systems have been reported to drastically alter the materials’ thermal transport properties,^3^, ^4^, ^5^, ^6^ depending on the geometry, dimensionality, crystallographic symmetry, and confinement. Interestingly, layered structures inherently exhibit structural anisotropy, a feature that can be detrimental or desirable depending on the application.^7^ In particular, when polymer films are filled with nanosheets of huge aspect ratio, the resulting nanocomposite properties ought to be exceedingly anisotropic. Yet, only effective material properties such as electrical or thermal conductivity have been reported in a direction‐dependent manner. Direction‐dependent mechanical properties, which fundamentally translate into thermal transport properties are still missing. Strong anisotropies in hybrid materials are preferentially achieved at small stacking periodicities5b or by combining components with a large property contrast.^8^

For a thorough characterization of such nanosheet/polymer stacks (also known as “nacre‐mimics”^9^), macroscopically oriented and homogeneous systems are paramount. Such ideal model system should also exhibit translational crystallographic symmetry, tunability, and strong anisotropy. Direction‐dependent studies benefit significantly from the availability of various light‐scattering methods, rendering a transparent filler such as the synthetic clay hectorite with a mica‐type structure desirable. For fundamental investigations of elastic properties, Brillouin light spectroscopy (BLS) has established itself as a technique of choice, as it allows for microscopic observations of high frequency (GHz) dynamics, at which viscoelasticity effects are usually negligible.^10^ On the other hand, lock‐in thermography and photoacoustic techniques have been proven reliable in accessing the in‐plane and cross‐plane thermal conductivities of thin films.^11^

Here, we show for the first time the complete mechanical properties of clay/polymer Bragg stacks that are fabricated using a uniquely defined, scalable spray‐coating process meeting all aforementioned specifications of a suitable model system. We, therefore, introduce 1D hybrid Bragg stacks based on nacre‐mimetic clay/polymer with small stacking periods and large property contrast. These Bragg stacks are scalable in both lateral extension and thickness, and they are macroscopically oriented. The fully controlled microstructure allows a detailed orientation dependent characterization of the thermal and mechanical properties. We couple the thermal and mechanical analyses to achieve an in‐depth understanding of the interplay between the thermal conductivities and mechanical moduli in a direction‐dependent manner. The extreme confinement of polymer between the clay sheets further prompts a question regarding the validity of continuum mechanics that we also address. The combination of unique hybrid materials and advanced characterization techniques provides an unprecedented insight into the physics of direction‐dependent nanomechanical and thermal transport properties in strongly anisotropic materials with polymer confinement.

## Results and Discussion

**Hybrid Bragg stacks with extreme polymer confinement**. The Bragg stacks comprise synthetic clay sodium fluorohectorite (Hec, [Na_0.5_]^inter^[Mg_2.5_Li_0.5_]^oct^[Si_4_]^tet^O_10_F_2_) and polyvinylpyrrolidone (PVP, *M*_w_=40 000 g mol^−1^). Like layered titanates^12^ and antimony phosphates,^13^ Hec belongs to a handful of compounds showing a rare phenomenon of osmotic swelling.^14^ In contrast to mechanical exfoliation by for example, sonication in the liquid phase,^15^ osmotic swelling is a thermodynamically favored, repulsive process,^16^ allowing for complete and gentle delamination that preserves the diameter of the parent crystals. In general, exfoliation describes the process of slicing tactoids into thinner stacks, whereas by delamination, the layered material is exfoliated to the level of individual single nanosheets.^17^ For Hec, nanosheets with a thickness of 10 Å and a median diameter of 20 μm (Figure S1) are obtained by simply immersing the material into deionized water.^18^ Phase purity and a homogeneous charge density guaranteeing a uniform intracrystalline reactivity are prerequisite for such a well‐controlled delamination. For Hec this is achieved by long‐term annealing, while less uniform natural or other synthetic clays commonly applied for nacre‐mimics comprise mixtures of auxiliary minerals, mono‐, few‐ and multilayer stacks.^18^

Because of the large aspect (diameter to thickness) ratio, polar rotation of the nanosheets in suspension is hindered, leading to parallel nanosheets after osmotic swelling. Even dilute (<1 vol. %) suspensions of Hec represent nematic phases.^19^ The parallel pre‐orientation of adjacent nanosheets in the highly swollen dispersion is indispensable for the fabrication of homogenous and periodic Bragg stacks via spray coating. Similar to titanate nanosheets,^20^ Hec nanosheets adopt this cofacial arrangement due to strong electrostatic repulsion with inter‐nanosheet distances exceeding 50 nm. Polymers can easily diffuse into these spacious galleries. By mixing Hec suspensions with varying aliquots of an aqueous PVP solution, we obtained perfectly homogeneous, nematic dispersions, as evidenced by small‐angle X‐ray scattering (SAXS) measurements (Figure S2).

Through spray coating of dilute nematic mixtures of high‐aspect‐ratio Hec nanosheets with PVP (1–2 wt % total solid content, see Section S1) highly coherent Bragg stack films with tunable gallery spacings are fabricated.^21^ The transverse flexibility of clay monolayers^22^ and their large aspect ratio are essentials assuring the high degree of precision obtained in the self‐assembly.^23^ Both, all nanosheets and the macroscopic film are aligned parallel to a polyethylene terephthalate substrate. The microscopic orientation of the Hec nanosheets prescribes the macroscopic film orientation, which is prerequisite for the direction‐dependent measurements. The macroscopic film orientation is, consequently, equivalent to the microscopic polymer/clay direction and allows using far‐field and integrating characterization techniques to reveal direction‐dependent properties. After drying, self‐supporting hybrid films with lateral extensions of several square centimeters are peeled off the substrate and used in the BLS and thermal conductivity measurements. Only by generating a nematic phase consisting of a homogeneous mixture of large aspect ratio and flexible nanosheets allows for fabrication of large area, self‐standing 1D single crystals referred to in literature as Bragg stacks or smectic films.^23^ Furthermore, appropriate processing like spray coating fostering the thermodynamic equilibration of the hybrid structure during drying has to be employed.

In total, we prepared six samples: pure polymer, pure Hec, and four hybrid Bragg stacks, which are denoted as Hec0/PVP100, Hec100/PVP0, Hec23/PVP77, Hec31/PVP69, Hec40/PVP60, and Hec51/PVP49, respectively. Here, the numbers indicate the volume fractions (vol. %) of Hec and PVP, as confirmed by thermogravimetric analysis (Table S1 and Figure S3).

**Figure 1:**
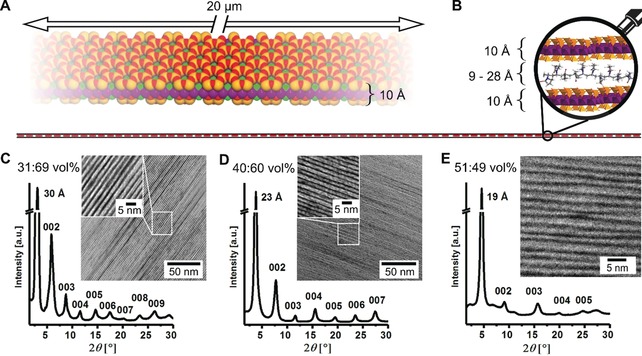
Schematic and microscopic images of ultra‐anisotropic and extremely confined Hec/PVP Bragg stacks. A) Space‐filling model of one single Hec nanosheet emphasizing the anisotropy of the nanosheet and the corrugation of the clay nanosheet allowing for interdigitation with PVP. B) True to scale schematic of the pronounced structural anisotropy. The ultra‐high‐aspect‐ratio nanosheets stretch from left to right and have lateral dimensions much larger than the length of the PVP polymer chains. The gallery height is on the order of magnitude of the molecular dimensions. C,D) XRD patterns of Hec31/PVP69 and Hec40/PVP60 (defect‐free materials) showing intense *001*‐reflections and a rational series of basal reflections up to the ninth order. The cross‐sectional TEM images show exceptionally periodic homogeneity of these hybrid films over large length scales. E) XRD patterns and cross‐sectional TEM image of Hec51/PVP49 displaying a random stacking of two gallery heights.

The Hec surface is corrugated (Figure 1 A) allowing for interdigitation and anchoring of PVP chains. Such interdigitation has been documented for intercalated molecular moieties, where structures based on single crystal data refinement are available.^24^ An in‐scale impression of the ultra‐high aspect ratio provided by the Hec nanosheet gallery is shown in Figure 1 B, where the length of the line corresponds to the typical lateral size of a clay nanosheet, and the thickness of the line to the height of a Hec/PVP/Hec layer. The magnifying lens highlights the extreme polymer confinement in the cross‐plane direction. The perfect homogeneous arrangement of Hec nanosheets and PVP is demonstrated by TEM and SEM images over different dimensions (Figure 1 C–E, Figure S7). Note that the lateral dimensions of the Hec nanosheets are much larger than the typical persistence and even contour lengths of the PVP chains. While for the polymer chains the Hec nanosheet confinement appears infinite, at the length scale of the Bragg‐stack films extending over tens of centimeters, they are of course finite. At the magnification where single 1 nm thick nanosheets are observable (Figure 1 C–E, Figure S5), the occurrence of nanosheet edges is very rare (fewer than one per 2500 nm^2^). Careful inspection, however, reveals few (Figure S6) of these nanosheet edges. The clay nanosheets show in‐plane crystalline order resembling the structure of mica. While mica possesses 3D crystalline order, our nanocomposite films belong to the transversely isotropic symmetry class, because the adjacent Hec nanosheets are positioned randomly in the lateral direction. However, all hybrid films show translational crystallographic symmetry along *001* (the cross‐plane direction), as indicated by several orders of Bragg reflections (Figure 1 C–E and Figure S5). By varying the PVP content, the basal spacing was tuned in the range from 19 to 38 Å, leading to PVP layer thicknesses ranging from 9 to 28 Å. For all samples, the gallery height is, therefore, significantly smaller than the PVP chains’ radius of gyration (*R*_g, PVP_≈15 nm^25^), implying strong polymer confinement.

In contrast to known nanocomposite films,9b, 26 Hec and PVP are thermodynamically miscible over a wide range of compositions. This miscibility is a prerequisite for tuning the basal spacing over a wide range with angstrom precision, which typically is only observed when vapour‐phase deposition techniques are applied.5a The miscibility is also reflected in an agreement of the basal spacing observed by X‐ray diffraction (*d*_XRD_) with the nominal values calculated based on the Hec and PVP volume fractions (*d*_nominal_, Table S1). To the best of our knowledge, our Hec/PVP Bragg stack films are the first of its kind showing such an agreement. Because the polymer confinement, however, is getting to the point where the gallery height is on the order of the size of an individual polymer chain, it is not possible to vary the gallery height continuously but only in incremental steps that relate to the diameter of the polymer chain. Consequently, only discrete polymer volume fractions lead to essentially defect‐free Bragg stacks, as seen in Hec40/PVP60 and Hec31/PVP69. XRD patterns reflect this with a rational *001*‐series, where the average basal spacing (*d*_XRD_) calculated from individual reflections shows a low coefficient of variation with the reflection peaks being sharp and intense (Table S1). The two gallery heights of the two defect‐free hybrid materials (1.3 nm for Hec40/PVP60 and 2.0 nm for Hec31/PVP69) might be attributed to the elliptical nature of PVP chains with principle axes of 1.0 nm and 1.3 nm (Figure S4 A). The observed gallery heights correspond to a monolayer with the longer principal axis (Figure S4 B) oriented perpendicular to the Hec nanosheets and a bilayer with the longer principle axis (Figure S4 C) lying in the plan of the Hec nanosheets, respectively.

In the two cases where the volume ratios do not happen to match (Hec23/PVP77 and Hec51/PVP49), the miscibility is nevertheless assured at small length scale by random interstratification of two gallery heights (Figure 1 E; transmission electron microscopy (TEM) close‐up), and the coefficient of variation of the *001*‐series increases^27^ (Table S1) with the reflection peaks being less intense (Figure 1 E and Figure S5).

**Figure 2:**
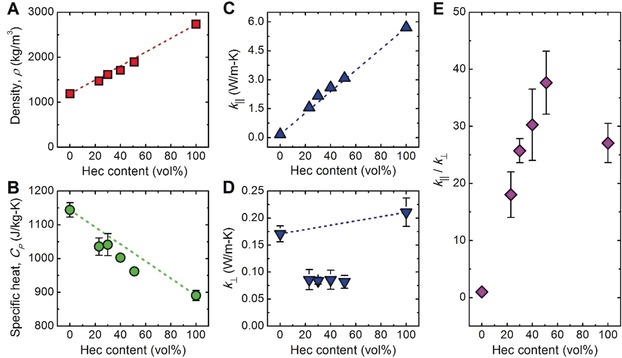
Direction‐dependent thermal conductivities of Hec/PVP hybrid Bragg stacks. A) Film density, B) specific heat, and effective C) in‐plane and D) cross‐plane thermal conductivities, as a function of the Hec volume fraction. The red, green, and blue dashed lines in (A)–(D) show linear trends based on a simple mixing model, *A*(*x*)=(1−*x*)*A*(0 %)+*xA*(100 %), where *A* represents *ρ, C_p_, k*_∥_, or *k*_⊥_, and *x* denotes the Hec volume fraction. E) Ratio of the in‐plane to cross‐plane thermal conductivities vs. the Hec volume fraction. For clarity, error bars smaller than the symbol size are not shown.

**In‐plane and cross‐plane thermal conductivities**. The in‐plane and cross‐plane thermal conductivities of the Hec/PVP hybrid Bragg stacks were characterized by lock‐in thermography and photoacoustic measurements,^11^, ^28^ respectively. Since the density, *ρ*, and specific heat, *C*_p_, are prerequisites for the thermal conductivity analysis, they were also determined experimentally by using helium pycnometry and differential scanning calorimetry (DSC) (Section S2), respectively. As the Hec volume fraction increases from 0 to 100 %, the density increases from 1190 to 2730 kg m^−3^ (Figure 2 A). This is well captured by a volume‐fraction‐based mixing model (dashed line in Figure 2 A). Correspondingly, the specific heat decreases from 1140 to 890 J kg^−1^ K^−1^ (Figure 2 B), which also follows the prediction by an effective medium model (Figure S8 B). Both analyses indicate that despite the extreme polymer confinement the properties of the hybrid stacks could be described by linearly interpolating the properties of the two bulk constituents. The polymer confinement, however, leads to a significant increase in the glass transition temperature (*T*_g_) of PVP, with no discernable *T*_g_ below 250 °C even at the lowest Hec composition (Figure S8 A). Expectedly, the thermal conductivity of the Bragg stacks strongly depends on the direction. The in‐plane thermal conductivity achieves its maximum, *k*_∥, max_=5.71 W m^−1^ K^−1^, in Hec100/PVP0, which is even higher than typical in‐plane thermal conductivities of natural micas (Figure 4 A).^29^ The lower end is given by the isotropic thermal conductivity of Hec0/PVP100, i.e., *k*_∥, min_=0.17 W m^−1^ K^−1^ (only determined by photoacoustic characterization). The four hybrid Bragg stacks have in‐plane thermal conductivities between these limiting values following a parallel mixing model (Figure 2 C).^30^ This is again surprising, as it implies that the confinement of PVP has no effect on the in‐plane thermal conductivity of the Hec/PVP hybrid stacks compared to bulk PVP. The cross‐plane thermal conductivity exhibits a broad minimum at *k*_⊥_≈0.09 W m^−1^ K^−1^, which is comparable to previously reported data for organoclay laminates.5b The deviation of the cross‐plane thermal conductivities from an effective medium behavior (Figure 2 D, dashed line) could be attributed to the Hec/PVP interfaces, which are the dominating contributors to the cross‐plane thermal resistance, as discussed below. The thermal conductivity anisotropy, *k*_∥_/*k*_⊥_, depends strongly on the hybrid composition, attaining a maximum of 38 in Hec51/PVP49 (Figure 2 E). We note that this anisotropy is exceptionally high for electrically insulating hybrid materials^31^ and outperforms natural nacre by a factor of ≈20.^32^ All in all, the structural perfection of the pure components and hybrid Bragg stacks translate into a record‐high in‐plane thermal conductivity and thermal transport anisotropy.

**Anisotropic mechanical properties**. The unique macroscopic orientation in the hybrid Bragg stacks allows us to track down the origin of their high thermal conductivity anisotropy by measuring their full mechanical tensors. The measurements were conducted by using BLS, which probes the phonon wave vector, **q**, dependent sound velocity, *v*, through inelastic light scattering by thermally excited, high frequency (GHz) phonons.^10^ Since the hybrid Bragg stacks are transversely isotropic, only **q** vectors in a single plane containing the symmetry axis have to be considered. For such a **q** vector, the direction can be denoted by *α*, the angle between **q** and the normal to the sample film, and because of symmetry *α* can be restricted in the range from 0° to 90°. The measurements corresponding to *α*=0°, 0° < *α*<90°, and *α*=90° were conducted in the reflection, backscattering, and transmission scattering geometries, respectively, while the polarization of the phonon mode was selected using different incident and scattered light polarization configurations (e.g., VV for quasi‐longitudinal (Q‐L) and quasi‐transverse (Q‐T) modes, and VH for a pure‐transverse (P‐T) mode).^33^ This flexibility of accessible parameters makes BLS particularly suitable for characterizing anisotropic or crystalline structures, as demonstrated in previous experiments on mica crystals.^34^ Since this work is the first one to apply the BLS technique to a hybrid Bragg stack material, we briefly outline the BLS measurement and data analysis.

**Figure 3:**
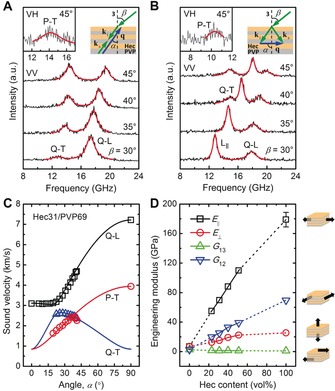
BLS measurements and strong mechanical anisotropy of Hec/PVP hybrid Bragg stacks. A,B) Polarized BLS spectra (anti‐Stokes side) of the Hec31/PVP69 hybrid stack film recorded in (A) the backscattering geometry with **q** forming a variable angle, *α*, with the normal to the sample film (top‐right inset to (A)) and (B) the transmission geometry with the phonon wave vector, **q**, directed in‐plane (*α*=90°; top‐right inset to (B)). **k**_i_ and **k**_s_ are the wave vectors of the incident and scattered light beams, respectively. *β* is the incident angle of the laser beam. The quasi‐longitudinal (Q‐L), quasi‐transverse (Q‐T), and in‐plane longitudinal (L_∥_) phonon modes are indicated in (A) and (B). The much weaker depolarized VH spectra of the pure transverse modes are shown in the top‐left insets. Notice the correspondence between the Q‐L and Q‐T modes in (A) and those in (B). C) Direction‐dependent sound velocities of the observed acoustic phonons in the BLS spectra of Hec31/PVP69. The three solid lines indicate theoretical representations (Equations (S6)–(S8)) of the experimental sound velocities of the three modes. D) Composition dependence of four engineering moduli. The moduli of the anisotropic hybrid films are extrapolated to those of the pure PVP and pure Hec films, as shown by the dashed lines. The four schematics beside (D) visualize the physical meanings of the corresponding moduli.

Consider Hec31/PVP69 as an example. A typical BLS spectrum from the reflection geometry (inset to Figure S14 A) displays a cross‐plane longitudinal (L_⊥_) mode in the VV polarization configuration (Figure S14 B). A typical BLS spectrum from the backscattering geometry (Figure 3 A, top‐right inset) depicts a Q‐L and a Q‐T mode in the VV polarization configuration (Figure 2 A) and a weak P‐T mode in the VH polarization configuration (Figure 3 A, top‐left inset). Comparatively richer information exists in a typical BLS spectrum from the transmission geometry (Figure 3 B, top‐right inset). In the VV polarization configuration, the BLS spectrum features an in‐plane longitudinal (L_∥_) and a Q‐L mode at a small laser incident angle, *β*, and an additional Q‐T mode at a large *β* (Figure 3 B). In the VH polarization configuration, a weak P‐T mode at all *β* (Figure 3 B, top‐left inset) is clearly resolved. In the transmission BLS spectra, the intensity ratio of the Q‐L and Q‐T peaks yields additional information (Figure S15 B) and the Q‐T mode intensity increases noticeably at higher Hec contents. By comparing the backscattering and transmission spectra, it becomes clear that the appearance of the Q‐L and Q‐T peaks in the latter (Figure 3 B) results from the scattering of the laser beam internally reflected on the sample's backside.^35^

Based on the frequency shift, *f*, from the BLS spectrum and the phonon wave vector, **q**, from the momentum conservation analysis, we calculated the sound velocity along a certain **q** as *v*=2π*f*/|**q**|. Whereas the reflection measurements give the *v*_Q‐L_ at *α*=0° and the transmission measurements result in the *v*_Q‐L_ and *v*_P‐T_ at *α*=90°, the backscattering measurements provide sound velocities for all the Q‐L, Q‐T, and P‐T modes at intermediate *α* angles, as limited by the sample's refractive index. These direction‐dependent sound velocities are reported in Figure 3 C for Hec31/PVP69; additional data for the other samples are shown in Figure S16 A–D. Since sound velocities are intimately related to the elastic stiffness tensor in the framework of the Christoffel equation,^36^ the availability of the former together with the measured sample densities (Figure 2 A and Table S4) enables unique determination of the latter. For a transversely isotropic material, the elastic stiffness tensor contains five independent elastic constants (e.g., *C*_11_, *C*_12_, *C*_13_, *C*_33_, and *C*_44_).^37^ Through χ^2^ fitting,^38^ we obtained the elastic stiffness constants (Figure S16 E and Table S4), which allow theoretical representation of the direction‐dependent sound velocities (solid lines in Figure 3 C and Figure S16 A–D) as well as determination of the engineering mechanical properties (Figure 3 D and Table S5). In addition, we analyzed the error bars (standard deviations) of the quantities according to principles of uncertainty propagation (Section S5).

This analysis provides the first direction‐dependent insights into the mechanical properties of hybrid Bragg stacks in general and of clay/polymer nanocomposites in particular. The Young's moduli, *E*_∥_ and *E*_⊥_, and torsional shear modulus, *G*_12_, all increase with increasing Hec volume fraction. The sliding shear modulus, *G*_13_, however, decreases from 2.6 GPa in Hec0/PVP100 to 1.0 GPa in Hec100/PVP0. A reduction in polymer chain entanglement upon confinement could be the cause of the decrease in *G*_13_.^39^ Since the elastic moduli of polymer nanocomposites depend on the specific filler‐polymer and polymer‐polymer interactions, a rationalization of the increase (*E*_∥_, *E*_⊥_, *G*_12_) or decrease (for *G*_13_) with Hec content would require computer simulations.^39^ All the mechanical moduli of the Bragg stacks display an effective medium behavior, assuming values between those of the two bulk components. We point out that even though the PVP chains are strongly confined between the adjacent Hec nanosheets (note *R*_g, PVP_>5(*d*_XRD_−*d*_Hec_)), bulk properties (e.g., *ρ*_PVP_, *ρ*_Hec_) are sufficient to fully capture the BLS measurements. As expected from the structural anisotropy, the Young's moduli exhibit large differences between the in‐plane and cross‐plane directions (Figure 3 D). As the Hec volume fraction increases from 0 to 100 %, the mechanical anisotropy ratio, *E*_∥_/*E*_⊥_, increases from 1 to 7. Concomitantly, the two characteristic Poisson's ratios, *ν*_31_ and *ν*_12_, vary in ranges of 0.02–0.05 (nearly zero or cork‐like values) and 0.34–0.41 (typical polymer values), respectively (Table S5). The reasonable values of the mechanical properties corroborate the validity of continuum mechanics at length scales of a few nanometers and in the presence of extreme polymer confinement.

**Figure 4:**
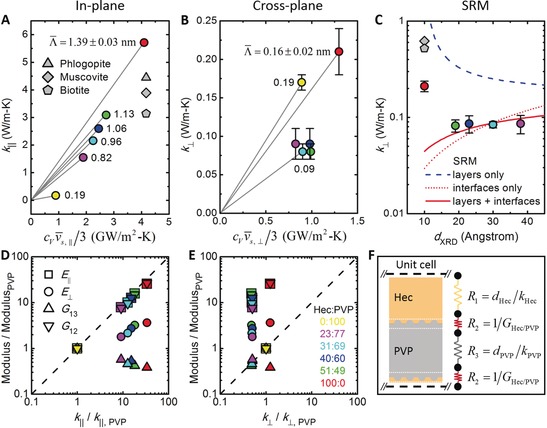
Analysis of anisotropic thermomechanical coupling in Hec/PVP hybrid Bragg stacks. A) Effective in‐plane thermal conductivity, *k*_∥_, vs. *C*_V_ *v̄*_s,∥_/3. The yellow, purple, cyan, blue, green, and red colored symbols represent the Hec0/PVP100, Hec23/PVP77, Hec31/PVP69, Hec40/PVP60, Hec51/PVP49, and Hec100/PVP0 samples, respectively. The phlogopite data point is from our additional measurements; muscovite and biotite data points are from reference.^29^ B) Effective cross‐plane thermal conductivity, *k*_⊥_, vs. *C*_V_ *v̄*_s,⊥_/3. In (A) and (B), the numbers beside the data points indicate the average phonon mean free paths, $\bar{\Lambda }$ (i.e., the slope of the gray lines). C) Effective cross‐plane thermal conductivity, *k*_⊥_, vs. the basal spacing of the Bragg stacks. The red solid line is a fit to the experimental data based on the series resistance model (SRM) shown in (F). As a comparison, the blue dashed line considers only the thermal resistances of the Hec and PVP layers, and the red dotted line considers only the thermal resistances of the Hec/PVP interfaces. D) Normalized mechanical moduli vs. normalized effective in‐plane thermal conductivity, *k*_∥_. The dashed line shows a direct correlation between the two axes with a power of one. E) Normalized mechanical moduli vs. normalized effective cross‐plane thermal conductivity, *k*_⊥_. In (D) and (E), the following values of the pure PVP film, *E*_∥_=*E*_⊥_=7.0 GPa, *G*_13_=*G*_12_=2.6 GPa, and *k*_∥_=*k*_⊥_=0.17 W m^−1^ K^−1^, are used as references in the normalization. F) A schematic of the SRM used to analyze the Hec/PVP interfacial thermal conductance.

In the last section, we summarize the new insights onto the anisotropic thermoelasticity that can be gained from this wholistic analysis. We firstly exploit the directly measured direction‐dependent sound velocities, and secondly correlate the derived mechanical moduli to the direction‐dependent thermal conductivities. We first apply a kinetic theory model, *k*=*C*_V_ *v̄*_g_ $\bar{\Lambda }$ /3 to estimate the average phonon mean free path $\bar{\Lambda }$ along different directions in the Bragg stacks.^40^ We use *C*_V_=*C*_P_ and *v̄*_g_=*v̄*_s,∥_=(*v*_Q‐L,∥_+*v*_Q‐T,∥_+*v*_P‐T,∥_)/3; a similar analysis is done for *k*_⊥_. The in‐plane $\bar{\Lambda }$ strongly depends on the hybrid composition, ranging from 14 Å for Hec100/PVP0 to 2 Å for Hec0/PVP100 (Figure 4 A). We note that these $\bar{\Lambda }$ values significantly underestimate the presence of longer ranged phonons, which are typically better described by a phonon mean free path accumulation function.^40^, ^41^ It is well known that thermal transport involves phonons over a wide range of frequencies which have different specific heat capacities, group velocities, and mean free paths. The underestimated $\bar{\Lambda }$ in our analysis could be attributed to the overestimated *v̄*_g_ from the BLS measurements, which mainly characterizes the propagation speed of a small fraction of the low frequency (long wavelength) phonons. These low frequency phonons carry only a negligible fraction of the overall heat. For the in‐plane direction, the lateral size of the Hec nanosheets by far exceeds the average phonon mean free path. Hence, the high in‐plane thermal conductivities are governed by the intrinsic material properties, not by the presence of grain boundaries between the aligned Hec nanosheets. The complementary analysis for *k*_⊥_ demonstrates a strong reduction of $\bar{\Lambda }$ down to less than 1 Å (Figure 4 B) with no discernible composition dependence along the cross‐plane direction. Interfacial effects apparently dominate the thermal transport in this direction, which is better analyzed using a series resistance model (SRM, Figure 4 C,F) as outlined by Losego et al.5b The fitted value of the interfacial conductance, *G*_Hec/PVP_=89±8 MW m^−2^ K^−1^ (see Section S4), falls well into the range of reported values for other inorganic/organic interfaces.2a, 42 The intercalation of PVP between the clay sheets leads to a strong reduction of the interfacial conductance, which is *G*_Hec/Hec_=219±28 MW m^−2^ K^−1^ for the pure hectorite.

We next address the correlation between the anisotropic mechanical moduli and thermal conductivities. Two distinct conclusions can be drawn. (i) In the direction parallel to the Hec nanosheets, a correlation between the thermal conductivity and all mechanical moduli is found. Along this direction the phonon mean free path is considerably shorter than the typical lateral size of a Hec nanosheet, rendering grain boundary effects insignificant. The influence of *E*_∥_, *E*_⊥_, and *G*_12_ on the thermal transport dominates over *G*_13_ since the former moduli show a direct relation to the in‐plane thermal conductivity. *E*_∥_ and *G*_12_ show a power scaling law close to one (0.93) between in‐plane thermal conductivity and modulus (*E*_⊥_ scales with 0.38). Whereas we find a clear correlation between the moduli and the thermal conductivity, we cannot deduce which change in mechanical modulus causes which effect to the thermal transport. The applicability of a simple mixing model along the parallel direction as outlined in Figure 2 A–C is certainly surprising in view of the strong polymer confinement effect on the glass transition (Figure S8). (ii) In the direction perpendicular to the Hec nanosheets, the phonon mean free path is comparable to the periodicity of the Bragg stacks. Here, the composition dependence of the mechanical properties does not influence the reduction of the cross‐plane thermal conductivity (vertical spread of the data points in Figure 4 E). Thus, changes to the gallery height are insignificant, which indicates the overwhelming contribution of the interfacial conductance. Considering changes to the pure components we find that reducing the sliding shear modulus *G*_13_ decreases the cross‐plane thermal transport properties of the polymer component. The reduction in *k*_⊥_ of the hybrid stacks relative to pure hectorite correlates to losses in *E*_∥_, *E*_⊥_, and *G*_12_ that apparently counteract the increase in *G*_13_. Overall, the mechanical and thermal properties are uncorrelated along the perpendicular direction, and the thermal transport is governed by the Hec/PVP interfaces.

## Conclusion

In conclusion, fully delaminated hectorite platelets can be processed into hybrid Bragg stacks with unique properties, with the polymer polyvinylpyrrolidone being the intercalated second component. Such long‐range 1D ordered materials become accessible by simply spray coating the desired nematic dispersions of adjusted volume fractions, which at the same time controls the periodicity of the hybrid stacks down to the angstrom level. The macroscopic lattice alignment enables the determination of direction‐dependent thermoelastic properties, which we assessed by thermal transport characterization techniques and Brillouin light spectroscopy. We found a record‐high anisotropy between the in‐plane and cross‐plane thermal conductivities in clay/polymer hybrid materials. This is corroborated by the first report of direction‐dependent Young's and shear moduli that are also strongly anisotropic. The effective gallery spacing, density, specific heat, and in‐plane thermal conductivity were found to conform to composition‐dependent simple mixing models. Despite the nanometer‐level lattice periodicity and angstrom‐level polymer confinement, the Christoffel‐equation‐based model, derived in the framework of continuum mechanics, remains applicable for determining the anisotropic elasticity. Of general relevance is the direction dependency of the way that the mechanical moduli and thermal conductivities correlate. In the in‐plane direction, where grain boundaries are negligible relative to the phonon mean free path, *E*_∥_, *E*_⊥_, and *G*_12_ directly correlate with the in‐plane thermal conductivity. In the cross‐plane direction, where the phonon mean free path is comparable to the lattice periodicity, the thermal transport is governed by the clay/polymer interfaces. We are convinced that a wholistic understanding of direction‐dependent thermoelastic properties will have a broad impact on important applications such as electronic packaging and thermoelectrics. This contribution is only a first step towards this goal. More work needs to be done for the deterministic—maybe even independent—design of mechanical and thermal properties. Future studies should also address the role of enthalpic interaction at the clay/polymer interface, interdigitation of the confined polymer, size effects of the platelets, and other nanosheet materials.

## Conflict of interest

The authors declare no conflict of interest.

## Supporting information

As a service to our authors and readers, this journal provides supporting information supplied by the authors. Such materials are peer reviewed and may be re‐organized for online delivery, but are not copy‐edited or typeset. Technical support issues arising from supporting information (other than missing files) should be addressed to the authors.

Supplementary
