# Supplementary material for: Tunable Thermoelastic Anisotropy in Hybrid Bragg Stacks with Extreme Polymer Confinement
Source: Angew Chem Int Ed Engl. 2019 Dec 4;59(3):1286–94. doi: 10.1002/anie.201911546 (PMC6972559; doi:10.1002/anie.201911546)
Supplement: Supplementary file 1 — Supplementary [file ANIE-59-1286-s001.pdf]

Angewandte Chemie
Eine Zeitschrift der Gesellschaft Deutscher Chemiker

Supporting Information

Tunable Thermoelastic Anisotropy in Hybrid Bragg Stacks with Extreme Polymer Confinement

Zuyuan Wang, Konrad Rolle[+], Theresa Schilling[+], Patrick Hummel[+], Alexandra Philipp[+], Bernd A. F. Kopera, Anna M. Lechner, Markus Retsch, Josef Breu,* and George Fytas**

anie_201911546_sm_miscellaneous_information.pdf

Abstract: Controlling thermomechanical anisotropy is important for emerging heat management applications such as thermal interface and electronic packaging materials. Whereas many studies report on thermal transport in anisotropic nanocomposite materials, a fundamental understanding of the interplay between mechanical and thermal properties is missing, due to the lack of measurements of direction-dependent mechanical properties. In this work, exceptionally coherent and transparent hybrid Bragg stacks made of strictly alternating mica-type nanosheets (synthetic hectorite) and polymer layers (polyvinylpyrrolidone) were fabricated at large scale. Distinct from ordinary nanocomposites, these stacks display long-range periodicity, which is tunable down to angstrom precision. A large thermal transport anisotropy (up to 38) is consequently observed, with the high in-plane thermal conductivity (up to 5.7 W m^{-1} K^{-1}) exhibiting an effective medium behavior. The unique hybrid material combined with advanced characterization techniques allows correlating the full elastic tensors to the direction-dependent thermal conductivities. We, therefore, provide a first analysis on how the direction-dependent Young's and shear moduli influence the flow of heat.

DOI: 10.1002/anie.201911546

Table of Contents

Section S0. Summary of Experimental Section 3
Section S1. Sample preparation 4
Materials 4
Film preparation 4
Section S2. Thermal measurements 8
Helium pycnometry 8
Differential scanning calorimetry 8
Lock-in thermography 9
Photoacoustic method 12
Section S3. Brillouin light spectroscopy 14
Section S4. Evaluation of interfacial thermal conductance 19
Section S5. Uncertainty analysis 20
References 21
Author Contributions 21

Section S0. Summary of Experimental Section

Sample preparation. The synthetic clay sodium fluorohectorite (Hec, $[Na_{0.5}]^{inter}[Mg_{2.5}Li_{0.5}]^{oct}[Si_4]^{tet}O_{10}F_2$) was delaminated by immersing it into Millipore water (0.5 wt%). The aqueous PVP solution (1 wt%) was added in the desired weight ratio. The suspension was mixed for at least one day in an overhead shaker. The homogeneity of the suspension was crosschecked by SAXS measurements. Self-supporting films were prepared using a fully automatic spray coating system. Every spraying cycle is followed by a drying cycle of 90 s at a temperature of 55 °C. We prepared pure PVP, pure Hec, and four hybrid Hec/PVP films. The self-supporting films were characterized by thermogravimetric analysis, XRD, and TEM. Additional information about the sample preparation can be found in Section S1.

In-plane thermal conductivity measurements. Lock-in thermography measures the temperature spreading across the free-standing samples upon thermal excitation by a focused laser beam with a modulated intensity. To prevent convective heat losses, the experiments are conducted in a vacuum chamber. The amplitude and phase data are extracted from the radial temperature distribution. The thermal diffusivity is then fitted by the slope method for thermally thin films. With the density, determined by helium pycnometry, and the specific heat, determined by differential scanning calorimetry (DSC), the thermal conductivity can be calculated. More details are provided in Section S2.

Cross-plane thermal conductivity measurements. The photoacoustic method uses a modulated laser beam to periodically heat a transducer layer in intimate contact with the sample. The heat is converted into an acoustic wave propagating into a gas tight cell above the sample, which is filled with helium at 20 psi. A sensitive microphone detects the phase shift between the acoustic signal and the modulated laser by a lock-in amplifier. The frequency-dependent phase shift is then compared to a multilayer model, assuming one-dimensional heat transfer. Therefrom, the total layer resistance is obtained. With the film thickness determined by AFM, the effective thermal conductivity is calculated. More details are provided in Section S2.

Brillouin light spectroscopy (BLS). BLS measures the phonon dispersion, $\omega(\mathbf{q})$, by detecting the Doppler frequency shift, ω, of the inelastically scattered light by sound waves (“phonons”) with a wave vector, $\mathbf{q}$. We recorded BLS spectra utilizing three scattering geometries (transmission, reflection, and backscattering) and two polarization configurations of the incident (λ = 532 nm) and scattered light (polarized VV, depolarized VH), which allowed us to establish the nature of the observed phonons. We varied the incident angle to obtain the direction-dependent sound velocities necessary for the determination of the anisotropic elasticity. The elastic stiffness tensors were obtained from the representation of the direction-dependent sound velocities by the Christoffel equation assuming transverse isotropy. The characteristic Young’s moduli, shear moduli, and Poisson’s ratios of the Bragg stacks were subsequently calculated. More details can be found in Section S3.

Section S1. Sample preparation

Materials

The synthetic clay sodium fluorohectorite (Hec, $[Na_{0.5}]^{inter}[Mg_{2.5}Li_{0.5}]^{oct}[Si_4]^{tet}O_{10}F_2$) was synthesized via melt synthesis followed by long-term annealing, according to an established procedure. The material featured a cation exchange capacity of 1.27 mmol g^{-1} (References[1]). Polyvinylpyrrolidone (PVP; M_w = 40.000 g mol^{-1}) was provided by Sigma Aldrich.

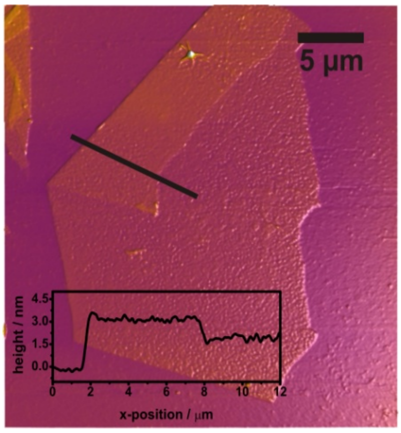

Figure S1. Atomic force microscopy image of a single delaminated Hec nanosheet. Reprinted with permission from Langmuir .[1b] Copyright 2019 American Chemical Society.

Film preparation

For the delamination, the synthetic Hec was immersed into Millipore water (0.5 wt%). The complete delamination was studied by small angle X-ray scattering (SAXS).[2] The aqueous PVP solution (1 wt%) was added in the desired weight ratio. The suspension was mixed for at least 1 day in the overhead shaker. Homogeneity of the suspensions was cross-checked by SAXS measurements. All SAXS data were measured using the small-angle X-ray system "Double Ganesha AIR" (SAXSLAB, Denmark). The X-ray source of this laboratory-based system is a rotating anode (copper, MicroMax 007HF, Rigaku Corporation, Japan) providing a micro-focused beam. The data were recorded by a position sensitive detector (PILATUS 300 K, Dectris). To cover the range of scattering vectors between 0.004-1.0 $Å^{-1}$, different detector positions were used. The measurements of the suspensions were done in 1 mm glass capillaries (Hilgenberg, code 4007610, Germany) at room temperature. To improve the detection limit of the in-house machine, the suspensions were first concentrated to ≈ 10 wt% by centrifugation at 10000 rpm for 1 hour. A rational basal series was found for all suspensions, indicating that all clay nanosheets were separated to exactly the same distance by water and PVP (Figure S2). Within experimental errors reaggregation to stacks of Hec can be excluded by the absence of a reflection typical for crystalline hydrated Hec phases at q = 0.65-0.41 $Å^{-1}$.

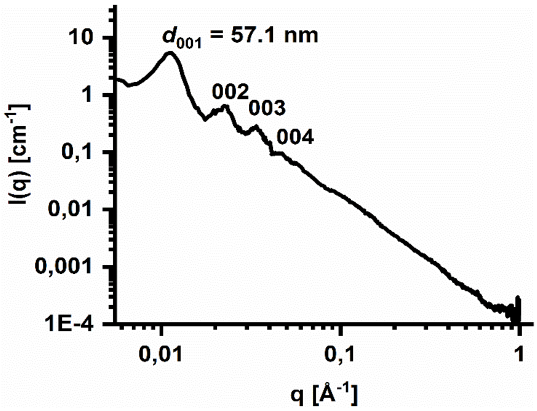

Figure S2. 1D SAXS pattern of the concentrated gel sample Hec40/PVP60.

The self-supporting films were prepared by spray coating. The fully automatic spray coating system was equipped with a SATA 4000 LAB HVLP 1.0 mm spray gun (SATA GmbH & Co. KG, Germany). Suspensions were sprayed on a corona-treated polyethylene terephthalate (PET) foil (optimont 501, bleher Folientechnik, Germany). The spraying and nozzle pressure were set constant at values of 2 and 4 bar, respectively. The round per flat fan control was set to 6 with a flow speed of 3 mL s^{-1}. The distance between the spraying gun and the substrate was 17 cm. The thickness of the suspension layer applied in one spraying step is ≈ 2 µm which corresponds to ≈ 20 nm dry nanocomposite film thickness. For drying the suspension layer, the sample is stopped under infrared lamps until evaporation of the solvent is complete. After every spraying cycle, a drying cycle of 90 s with a temperature of 55 °C took place. The spraying/drying cycle is repeated until the desired barrier film thickness of 50 µm is obtained. Afterwards, the film was dried at 100 °C for 3 days and peeled off from the PET foil to achieve self-supporting films. For characterization by photoacoustic analysis thinner films on the order of a few µm were spray coated onto clean 1 mm thick microscopy glass slides.
In total, we prepared six samples, which are denoted as Hec0/PVP100, Hec23/PVP77, Hec31/PVP69, Hec40/PVP60, Hec51/PVP49, and Hec100/PVP0, respectively (Table S1). Throughout the manuscript, all samples are referred to by the nominal volume fractions (vol%) of Hec and PVP, respectively. To rule out compositional changes during spray coating, these ratios were cross-checked (Table S1) by thermogravimetric analysis (TGA), using a Linseis STA PT 1600 (Linseis Messgeräte GmbH, Germany). Changes in mass observed upon heating in synthetic air up to 800 °C were attributed to the combustion of PVP.

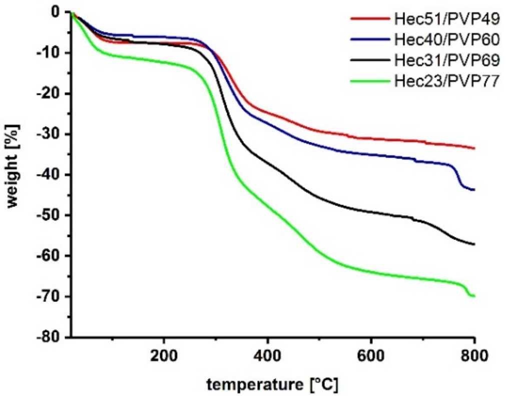

Figure S3. TGA curves of four hybrid Bragg stacks. The weight loss below 200 °C corresponds to the water.

X-ray diffraction (XRD) patterns for the films were recorded in Bragg-Brentano-geometry on an Empyrean diffractometer (PANalytical B.V.; the Netherlands) using Cu K_α radiation (λ = 1.54187 Å). The self-supporting films were placed on glass slides (Menzel-Gläser; Thermo Scientific). Prior to the XRD measurements, the samples were dried at 100 °C in a vacuum chamber for one week.

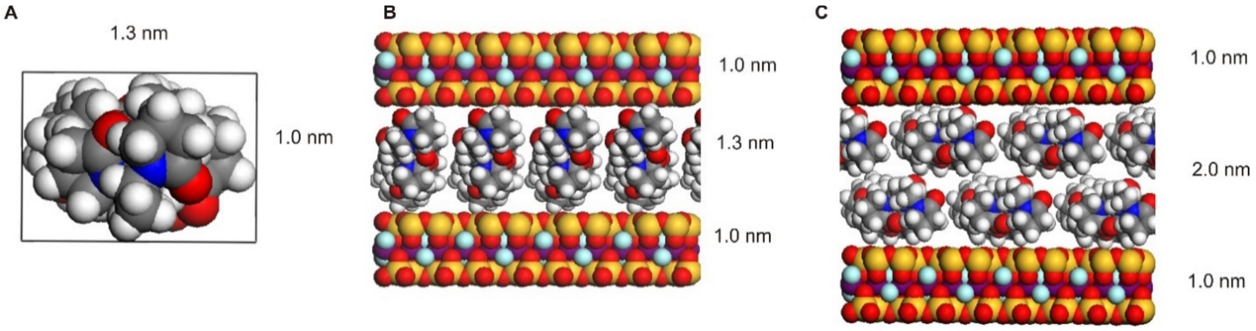

Figure S4. Space filling models. (A) PVP viewed along the polymer backbone chain. (B) Monolayer of PVP oriented with the longer principal axis perpendicular to the Hec nanosheet. (C) Bilayer of PVP lying in the plane of the Hec nanosheets.

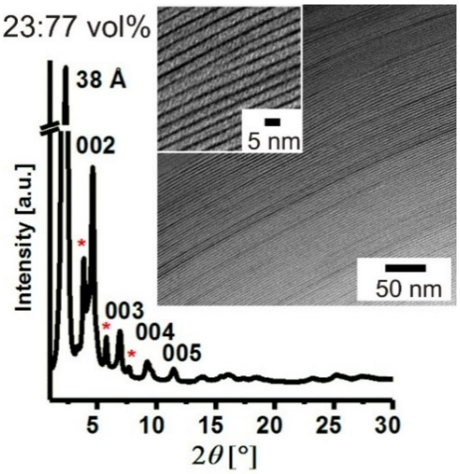

Figure S5. XRD pattern and TEM image of the nanocomposite film Hec23/PVP77. The red asterisks denote a second series of basal reflections of a minority phase (d = 2.3 nm).

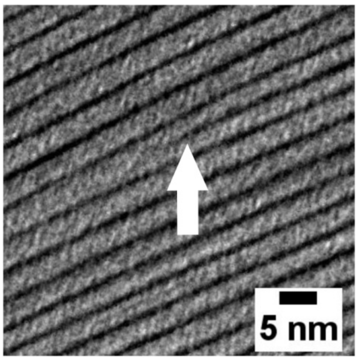

Figure S6. Lattice plane termination of a single Hec nanosheet.

As a measure of the quality of the one-dimensional crystallinity of the films, the coefficient of variation (CV) and the full width at half maximum (FWHM) were determined (Table S1). Large CV-values (≈ 3% [3]) and large FWHM indicate non-rationality of the diffraction pattern as caused by a random interstratification of different interlayer heights.
Assuming PVP and Hec densities of 1.2 g cm^{-3} and 2.7 g cm^{-3}, respectively, nominal d-spacings can be calculated for the various volume ratios ($d_{nominal}$ in Table S1).[4] They agree reasonably with those obtained from XRD measurements (d_{XRD} in Table S1). In analyzing the cross-plane thermal conductivity using the series resistance model, we used the d_{XRD} values.

Table S1. Overview of the structural and chemical characterization.

	nominal Hec:PVP ratio [wt%]	nominal Hec:PVP ratio [vol%]	PVP content [a] [wt%]	PVP content [b] [vol%]	$d_{nominal}$ [Å]	d_{XRD} [Å]	gallery height (PVP) [c] [nm]	CV [%]	FWHM [°2θ]
Hec100/PVP0	100:0	100:0	-	-	10	10	-	-	-
Hec51/PVP49	70:30	51:49	27	45	18	19	0.9	5.9	0.4
Hec40/PVP60	60:40	40:60	38	58	23	23	1.3	1.3	0.3
Hec31/PVP69	50:50	31:69	49	68	30	30	2.0	1.0	0.3
Hec23/PVP77	40:60	23:77	59	76	41	38	2.8	2.8	0.3
Hec0/PVP100	0:100	0:100	100	100	-	-	-	-	-

[a] determined by TGA; [b] calculated with the PVP content determined by TGA; [c] gallery height corresponds to the *d*-spacing minus the Hec platelet height of 1 nm.

Scanning electron microscopy (SEM) images were taken with a Zeiss LEO 1530 (Carl Zeiss AG, Germany) at an operating voltage of 3 kV. Cross sections were prepared by cutting with a razor blade. The samples with Hec were sputtered with 10 nm carbon.

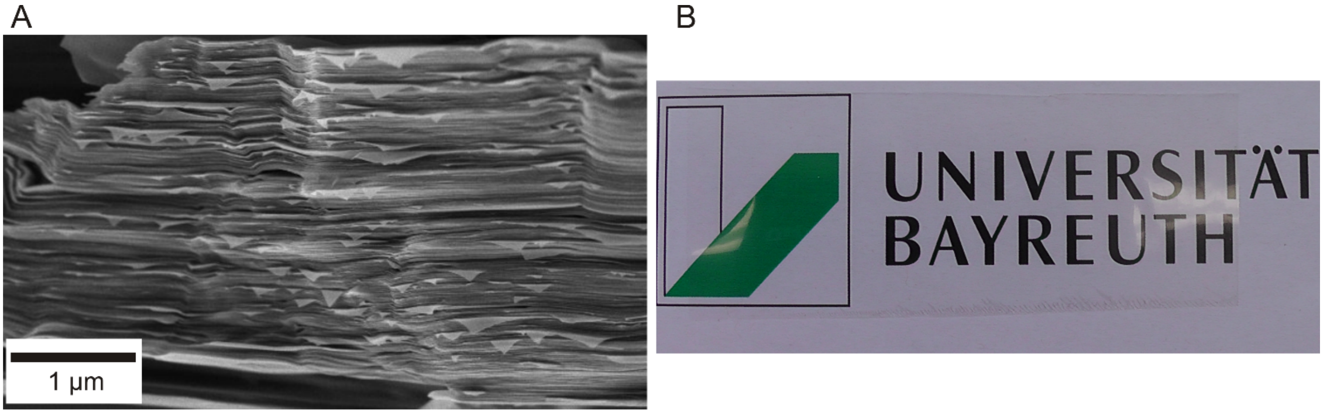

Figure S7. SEM image and photograph of the hybrid film. A) The SEM image displays the ordered arrangement of hectorite sheets at the macro-scale. B) As the hectorite platelets arrange highly ordered, light scattering is prevented and the films is transparent.

Transmission electron microscopy (TEM) images were taken on a JEOL JEM-2200FS (JEOL GmbH, Germany) at an acceleration voltage of 200 kV. Cross-section pictures of the self-supporting films were prepared with a Cryo Ion Slicer IB-09060CIS (JEOL, Germany).

Section S2. Thermal measurements

For the determination of the in-plane and cross-plane thermal conductivity, the density and the specific heat are needed. Therefore, Helium pycnometry and differential scanning calorimetry (DSC) were used. We determined the in-plane thermal diffusivity by lock-in thermography, and the cross-plane thermal conductivity by the photoacoustic method. Prior to the measurements, the samples were dried at 100 °C in a vacuum chamber for one week.

Helium pycnometry

The density of the samples was measured by helium pycnometry. Therefore, an Ultrapyc 1200e (Quantachrome Instruments) was used. Prior to each measurement the volume of the empty measurement cell was measured. Afterwards, small pieces of the free-standing films were weighed into the sample cell with a nominal volume of 1.8 cm^3. One hundred runs were performed to determine the volume of the films at room temperature. By knowing the mass (measured on a fine balance) and the volume, the density of the samples was calculated.

Differential scanning calorimetry

The specific heat was determined by DSC measurements according to the ASTM E1269 standard. The samples were freeze ground for better processability and contact to the DSC pans. The measurements were performed on a TA instruments Discovery DSC 2500. An isothermal step (1h, 100 °C) was conducted prior to the measurement to ensure dry conditions. Then two heating cycles were used and only the second cycle evaluated. The temperature profile ranged from -40 to 250 °C using a heating rate of 20 K min^{-1} with a nitrogen flow of 50 mL min^{-1}.

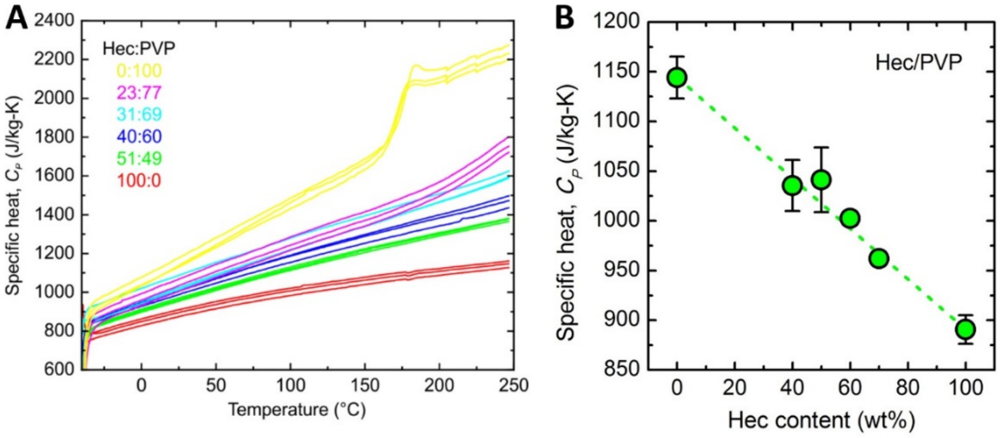

Figure S8. Temperature and composition dependencies of the specific heat. (A) Three samples per Hec/PVP composition were measured. The average specific heat at 25 °C was used to calculate the thermal conductivity. (B) The green dashed line shows the prediction by a simple mixing model, $C_P(x) = (1-x)C_P(0\%) + xC_P(100\%)$, with x being the hectorite weight fraction.

Lock-in thermography

The in-plane thermal diffusivity of free-standing Hec/PVP stack films was obtained by lock-in thermography (LIT). The self-built LIT set-up is shown in Figure S9.

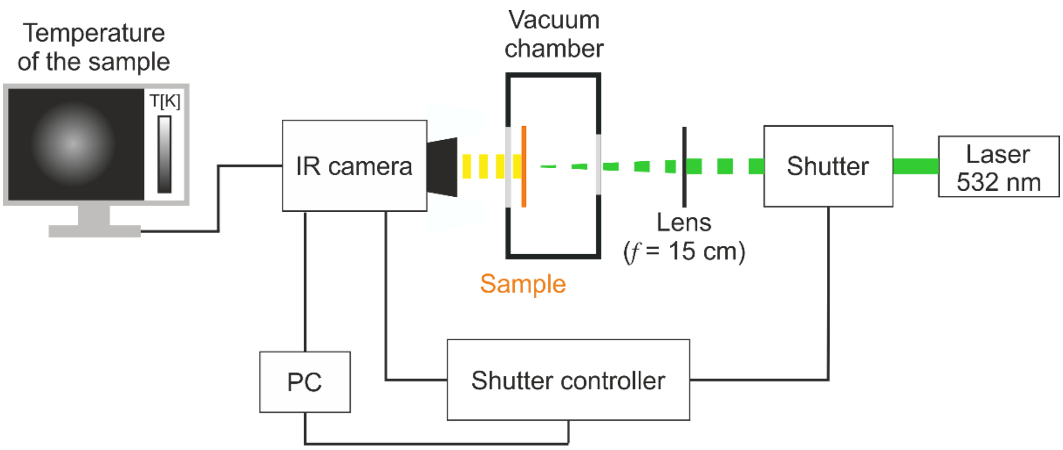

Figure S9. Scheme of the lock-in thermography set-up. The samples were measured in a vacuum chamber to avoid convective heat losses to the environment.

The sample is heated by a laser beam (Genesis MX 532-1000 SLM OPS, Coherent, λ = 532 nm) focused onto the sample surface by a lens of 150 mm focal length. The intensity of the laser is modulated using a shutter (SH05/M, Thorlabs) controlled by a shutter controller (SC10, Thorlabs). The emitted infrared (IR) radiation of the sample surface is detected by an Infratec VarioCAM HD research IR camera (spectral window: 7.5-14 µm). The camera is equipped with a close-up lense. In this configuration, the minimum spatial resolution is 29 µm (working distance: 33 mm). Since heat losses to the environment lead to an overestimation of the thermal diffusivity[5] all samples were measured under vacuum conditions (~3x10^{-3} mbar). Furthermore, all samples were coated with a 20 nm carbon layer for enhanced laser absorption. The coating of the sample was facing to the IR camera. LIT measurements were performed using Infratec's IRBISactiveonline software. Measurements were conducted at several lock-in frequencies between 0.219 and 1.765 Hz (depending on the Hec/PVP composition). Furthermore, each measurement was averaged over several (600-2000) lock-in periods to enhance the signal to noise ratio, with the first 60-100 periods being discarded. The software calculates automatically the amplitude and phase of the temperature oscillations (Figure S10). The red point marks the midpoint of the laser excitation and thus the midpoint of the radial distribution.

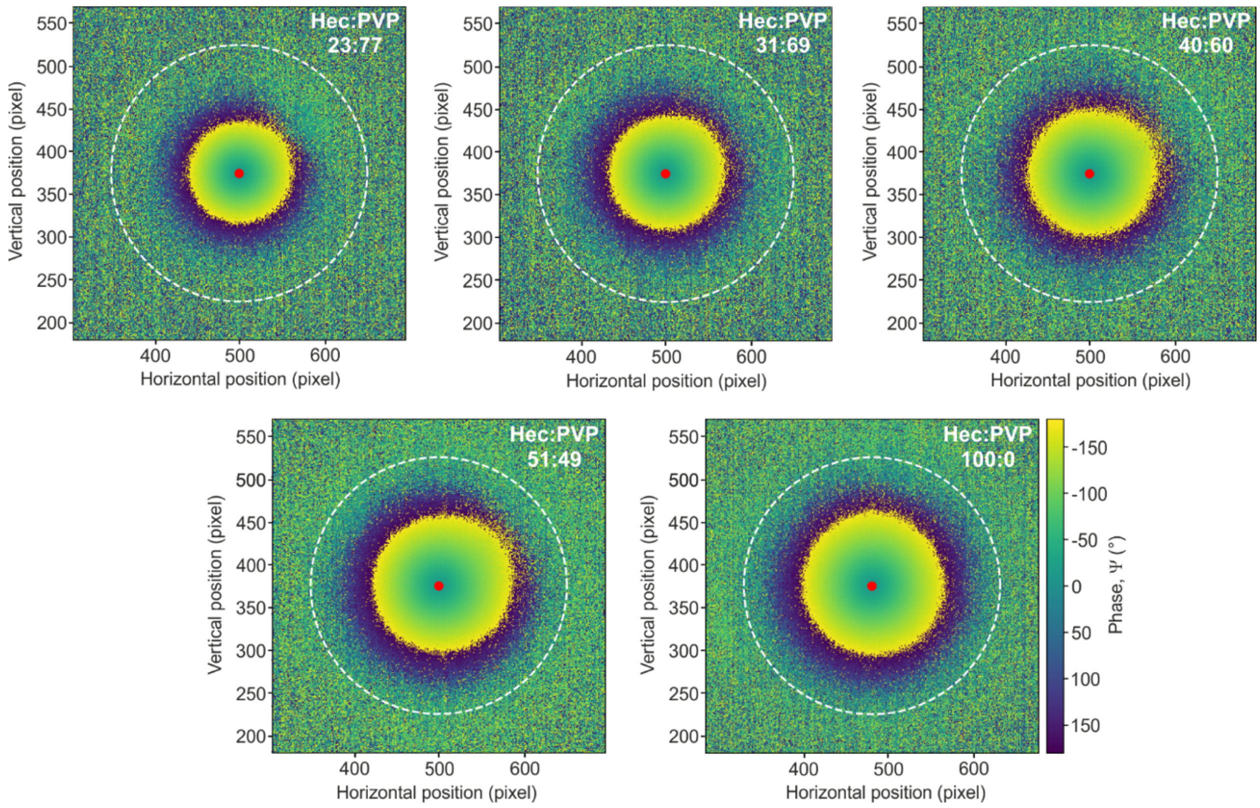

Figure S10. Exemplary two-dimensional phase images measured at a frequency of 1.111 Hz. The penetration depth of the temperature oscillations increases with increasing thermal diffusivity of the Hec/PVP films. The focal point of the laser is marked by a red point in the center of the IR image.

A self-written Python script is used to extract radial profiles for the phase and amplitdue images (Figure S11). The thermal diffusivity is calculated from the phase and amplitude slopes according to the slope method of a thermally thin film[5b]:

$$m_{\Psi} \cdot m_{\ln(T \cdot r^{0.5})} = \frac{\pi f_{\text{lock-in}}}{\alpha_{\|}} \quad \text{(S1)}$$

Here, m_{Ψ} is the slope of the linear relation of the phase and the radial distance r, $m_{\ln(T \cdot r^{0.5})}$ is the slope of the linear relation of the natural logarithm of the amplitude T multiplied by the square root of the radial distance r, $f_{\text{lock-in}}$ is the lock-in frequency, and $\alpha_{\|}$ is the in-plane thermal diffusivity.

Three films have been measured for each Hec/PVP composition. An average in-plane thermal diffusivity and a standard deviation were calculated for each composition, as summarized in Table S2.

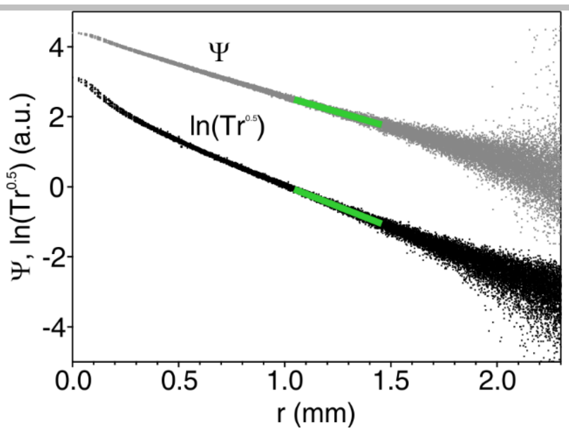

Figure S11. Exemplary phase Ψ and amplitude profiles. The green line indicates the region, where the linear fit was evaluated. This is sufficiently far away from the central excitation spot.

Table S2. In-plane thermal diffusivity values of Hec/PVP stack films. Three films per Hec/PVP composition were measured, based on which an average in-plane thermal diffusivity and a standard deviation were calculated.

Sample	In-plane thermal diffusivity ($mm^2\ s^{-1}$)
Hec23/PVP77	1.02 ± 0.04
Hec31/PVP69	1.28 ± 0.02
Hec40/PVP60	1.52 ± 0.04
Hec51/PVP49	1.69 ± 0.03
Hec100/PVP0	2.35 ± 0.03

The in-plane thermal conductivity was calculated from the in-plane thermal diffusivity ($\alpha_{||}$), density (ρ), and specific heat (C_P) as

$$k_{||} = \alpha_{||} \cdot \rho \cdot C_P \qquad \text{(S2)}$$

Photoacoustic method

The cross-plane thermal conductivity was determined by the photoacoustic method. It uses the photoacoustic effect to determine the thermal properties of a sample. Therefore, a modulated laser beam (λ = 488 nm) periodically heats the sample. For good absorption of the laser energy a thin Au transducer layer (~ 150 nm) was coated on the sample surface. For photoacoustic characterization, the samples were spray-coated on a glass substrate. The layout of the measurement cell above the sample is shown in Figure S12A. The gas tight cell is filled with a helium pressure of 20 psi. The microphone (Bruel&Kjaer, 4398-A-011) connected to the cell measures an acoustic wave, which is induced by the periodic heat conduction from the transducer layer surface to the gas phase. As shown in Figure S12B, the microphone is linked to a lock-in amplifier with integrated signal generator (Zurich instruments, HF2LI). The signal generator controls the electro-optic modulator (EOM, Conoptics, M25A) and therefore the frequency of the modulated laser beam. The acoustic signal detected by the microphone is then transferred into a phase and amplitude shift in relation to the modulation of the incident laser beam.

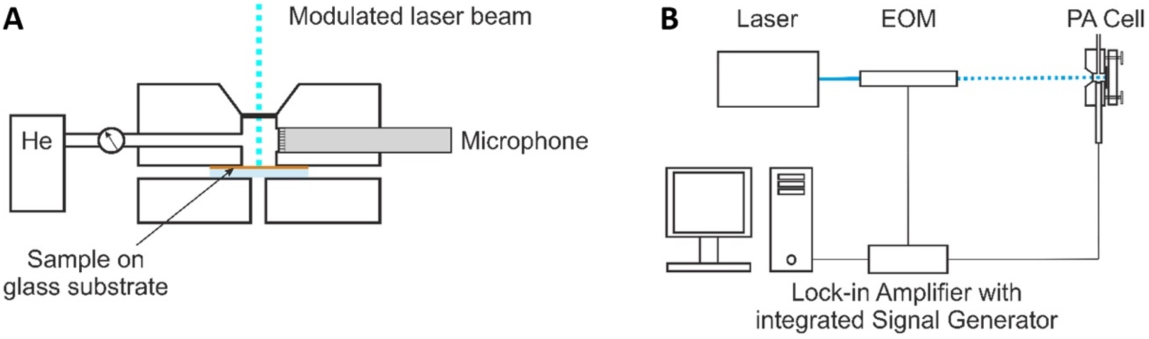

Figure S12. Scheme of photoacoustic measurements. (A) The photoacoustic cell. (B) The whole setup.

The phase shift is detected as a function of the frequency in a range from 110 Hz to 4000 Hz. The signal is then normalized with the phase shift signal of a thermally thick glass sample (1 mm) with known thermal properties. A representative frequency sweep is shown in Figure S13. The red line indicates the best fit according to the fitting procedure presented by Singh et al..[6] They used the generalized multilayer model of Hu et al.[7] assuming one-dimensional heat transfer. The unknown fitting parameters are the contact resistance between the gold layer and the sample, the thermal diffusivity of the sample, and the contact resistance between sample and substrate. For thin films the fit is not very sensitive to the individual parameters, but to the total layer resistance. Therefore, only the total layer resistance is reported. From the total layer resistance it is possible to calculate the effective thermal conductivity by dividing with the thickness. The thickness of the samples was determined by AFM measurements. The values of the total layer resistance and sample thickness are summarized in Table S3. For each Hec/PVP ratio samples with three different thicknesses were measured to exclude influences from the sample thickness.

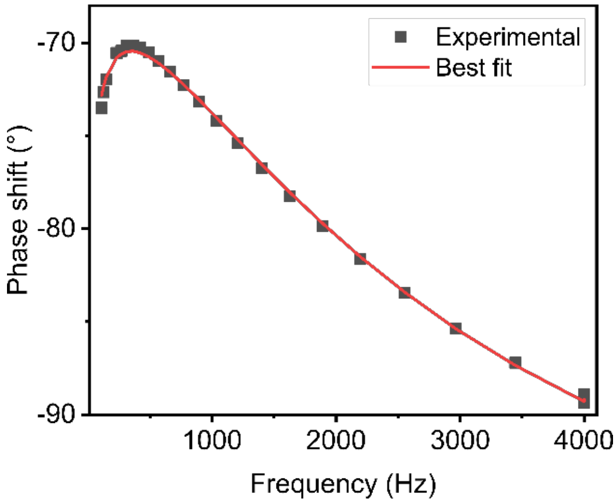

Figure S13. Normalized photoacoustic phase signal. Representative photoacoustic measurement of the Hec31/PVP69 sample with a thickness of 1.979 µm. The red line shows the best fit.

Table S3. Summary of the photoacoustic measurements. The total layer resistance, the thickness, and the resulting effective cross-plane thermal conductivity are given for each sample.

Sample	Total layer resistance [$mm^2\ K\ W^{-1}$]	Thickness [μm]	Effective cross-plane thermal conductivity [$W\ m^{-1}\ K^{-1}$]
Hec100/PVP0	1.58	0.28	0.177
Hec100/PVP0	2.55	0.595	0.234
Hec100/PVP0	4.34	0.94	0.217
Hec51/PVP49	3.69	0.255	0.069
Hec51/PVP49	7.74	0.722	0.093
Hec51/PVP49	11.53	0.957	0.083
Hec40/PVP60	6.54	0.463	0.071
Hec40/PVP60	14.03	1.139	0.081
Hec40/PVP60	18.96	1.987	0.105
Hec31/PVP69	8.20	0.744	0.091
Hec31/PVP69	17.58	1.382	0.079
Hec31/PVP69	24.06	1.979	0.082
Hec23/PVP77	8.81	0.568	0.064
Hec23/PVP77	17.45	1.677	0.096
Hec23/PVP77	26.37	2.568	0.097
Hec0/PVP100	2.26	0.351	0.155
Hec0/PVP100	4.50	0.792	0.176

Section S3. Brillouin light spectroscopy

Brillouin Light Spectroscopy (BLS) measures the phonon dispersion, i.e., the wave vector, **q,** dependent sound velocity, $v_i(\mathbf{q})$, by detecting the Doppler frequency shift, *f*, in laser light inelastically scattered by sound waves ("phonons"). Since the phonon modes in question are thermally populated, the Bragg condition for light scattering is satisfied independent of the scattering angle, in contrast to the kindred time-domain ("picosecond ultrasonic") techniques that require external phonon injection via special sample preparation. Since the latter typically involves deposition of non-transparent metallic transducer films, they also do not readily allow for transmission and backscattering measurements, further limiting the potential of these techniques for probing anisotropic materials.

Assuming a planar sample, BLS can be conducted in three scattering geometries: transmission, reflection, and backscattering. For angle-dependent measurements in the transmission and reflection geometries, the laser source (λ = 532 nm) was mounted on a goniometer and rotated around the sample, similar to a wide-angle X-ray (WAXS) setup. For the transmission geometry (top-right inset to Figure 3B), the propagation vector, **q,** of the sound wave lies in the plane of the sample and its modulus, $|\mathbf{q}|$, is independent of the refractive index according to $|\mathbf{q}|_{\parallel} = (4\pi/\lambda)\sin\beta$, with β being the light incident angle. For the reflection geometry, the Bragg condition is fulfilled for sound waves with **q** along the normal to the sample plane, so that $|\mathbf{q}|_{\perp} = (4\pi/\lambda)\sqrt{n^2 - \sin^2\beta}$, with *n* being the refractive index of the sample.[8] In order to find *n*, several points at different β were acquired, and then fit linearly; *n* was determined under the constraint that the fit has to pass through the origin (Figure S14A), and the obtained values are reported in Table S4. In contrast to the transmission geometry, the range of the dispersion relationship that can be scanned by varying $|\mathbf{q}|_{\perp}$ is more restricted due to refraction of the laser beam at the air-sample boundary. Finally, backscattering measurements can be conducted, where the incident and scattered light traverse the same path, and the sample is mounted on a rotating stage with angular gradation marks. Only a single dispersion point, corresponding to **q** directed opposite to $\mathbf{k}_i$ in the film and of a magnitude, $|\mathbf{q}| = 4\pi n/\lambda$, is accessible in the backscattering geometry, but all possible $\mathbf{q}/|\mathbf{q}|$ directions can be probed. Because backscattering measurements use the same lens for focusing the incident and collimating scattered light, the alignment of the setup is generally much easier, so that all the backscattering spectra were acquired with microscope objectives (typically 10x) to reduce the spectrum accumulation time.

From the given formulas, it is easy to see that the Brillouin frequency shift does not exceed $2nv/\lambda$, where *v* is sound velocity. These frequencies render viscoelasticity effects negligible and are detected using a high-resolution six-pass Tandem Fabry Perot (TFP) interferometer (JRS Instruments, Switzerland) optimized for the 1-50 GHz range. Values still further out on the dispersion relationship can be accessed using, for example, picosecond ultrasonic interferometry (PUI), but at the expense of great effort and without any gain in information for the problem at hand.[9] Finally, BLS also offers direct access to the shear moduli, simply by analyzing scattered light in different polarizations: VV (i.e., vertical incident and vertical scattered light) corresponds to quasi-longitudinal and quasi-transverse phonon modes, and VH (i.e., vertical incident and horizontal scattered light) to the pure-transverse phonon mode (for transversely isotropic samples, HV does not show a transverse peak). Before the BLS measurements, the samples were dried at 100 °C in a vacuum chamber for one week to remove any residual water content.

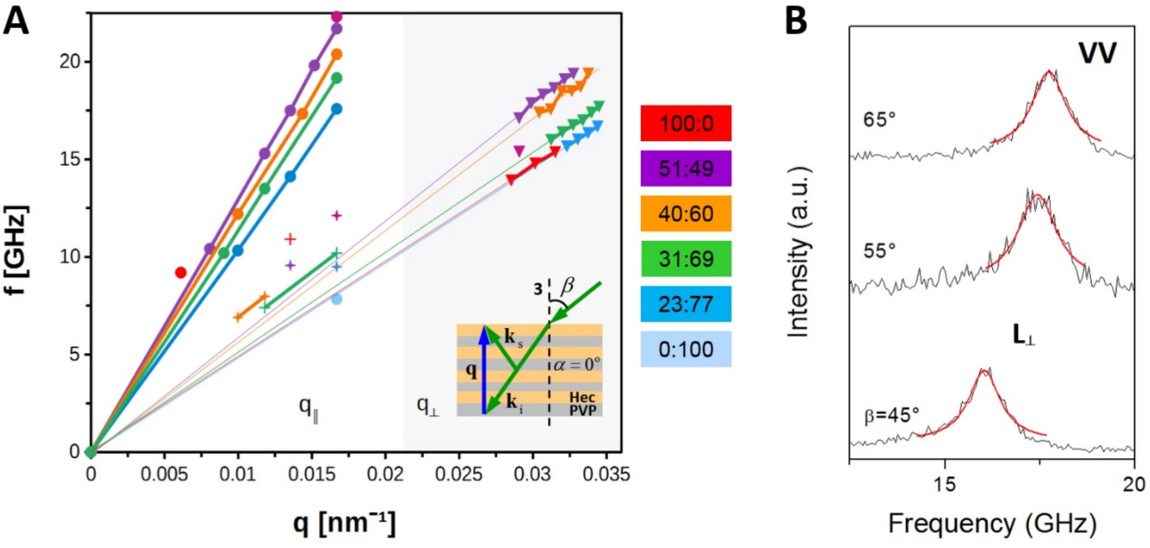

Figure S14. BLS experiments in the reflection geometry. (A) Dispersion (frequency vs. wave vector) of the longitudinal (L) and transverse (T) phonons in the hybrid stacks and the two constituent components (see the color code) obtained from the polarized (VV) and depolarized (VH) BLS spectra recorded in the reflection geometry (inset). (B) Typical VV spectra of the Hec31/PVP69 hybrid film at three laser incident angles.

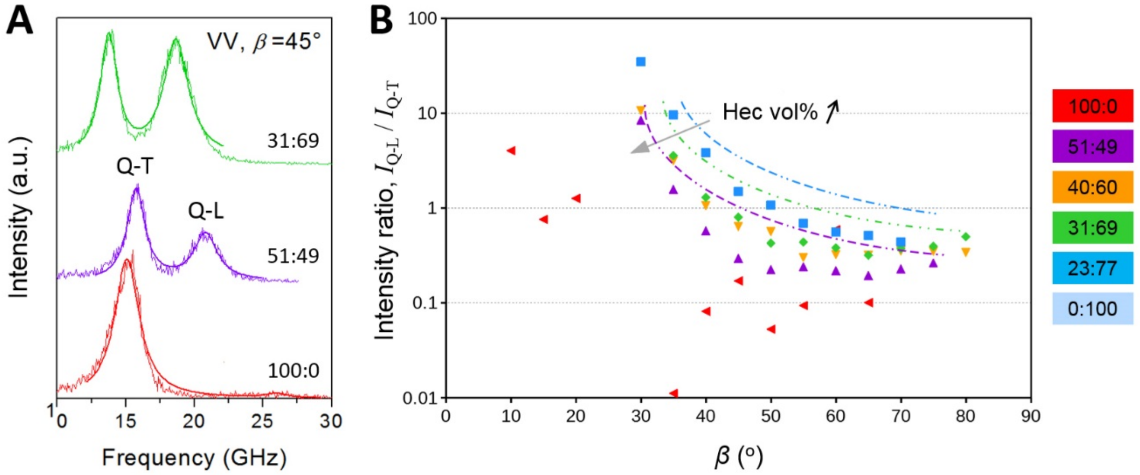

Figure S15. BLS spectra and peak intensity. (A) Polarized (VV) BLS spectra recorded in the backscattering geometry (inset to Figure 3A) for two hybrid stacks and the pure Hec film at an incident angle of 45°. (B) The intensity ratio of the Q-L and Q-T peaks in backscattering VV spectra vs. the laser incident angle.

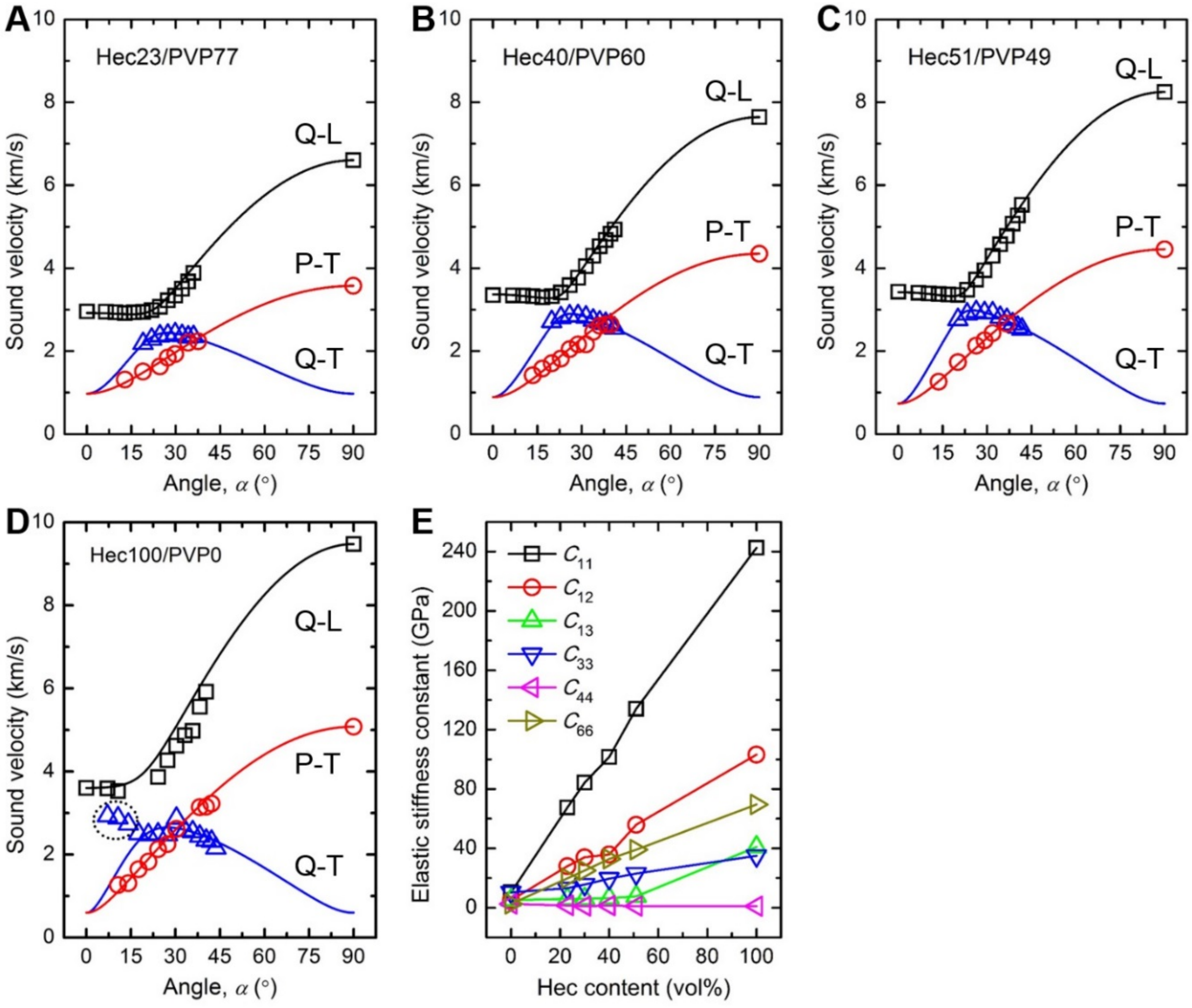

Figure S16. Direction-dependent sound velocities of hybrid stack films. (A) Hec23/PVP77, (B) Hec40/PVP60, (C) Hec51/PVP49, and (D) Hec100/PVP0 (the data points in the dotted circle were not used in the fitting). Q-L, P-T, and Q-T represent the quasi-longitudinal, pure-transverse, and quasi-transverse phonon modes, respectively. (E) Variation of the elastic stiffness constants with the Hec volume fraction.

Based on the BLS-measured direction-dependent sound velocities, we obtained the elastic tensor within the framework of the Christoffel equation.[10]

$$\begin{bmatrix} \Gamma_{11}-\rho v^2 & \Gamma_{12} & \Gamma_{13} \\ \Gamma_{21} & \Gamma_{22}-\rho v^2 & \Gamma_{23} \\ \Gamma_{31} & \Gamma_{32} & \Gamma_{33}-\rho v^2 \end{bmatrix} \begin{bmatrix} u_1 \\ u_2 \\ u_3 \end{bmatrix} = \begin{bmatrix} 0 \\ 0 \\ 0 \end{bmatrix}, \quad \text{(S3)}$$

where ρ is the density of the sample, v is the sound velocity, $\mathbf{u}=[u_1,\ u_2,\ u_3]^{\mathrm{T}}$ is the displacement vector, and Γ_{ik} (i, k = 1, 2, 3) is the Christoffel stress, which is defined as

$$\Gamma_{ik} = \sum_{j=1}^{3}\sum_{l=1}^{3} C_{ijkl} n_j n_l \, . \quad \text{(S4)}$$

Here, C_{ijkl} denotes an element of the 4th rank elastic tensor, and $\mathbf{n}=[n_1,\ n_2,\ n_3]^{\mathrm{T}}$ represents the phonon propagation direction.

For a transversely isotropic material, the elastic tensor, in the Voigt notation, has the following form.[11]

$$\mathbf{C} = \begin{bmatrix} C_{11} & C_{12} & C_{13} & & & \\ C_{12} & C_{11} & C_{13} & & & \\ C_{13} & C_{13} & C_{33} & & & \\ & & & C_{44} & & \\ & & & & C_{44} & \\ & & & & & C_{66} = \dfrac{C_{11} - C_{12}}{2} \end{bmatrix}, \quad \text{(S5)}$$

and contains five independent stiffness constants. After some algebra, the sound velocities of the Q-L, Q-T, and P-T modes along a direction defined by *α* can be represented as follows,

$$v_{\text{Q-L}}(\alpha) = \sqrt{\frac{-A_1 + \sqrt{A_1^2 - 4A_2}}{2\rho}} \quad \text{(S6)}$$

$$v_{\text{Q-T}}(\alpha) = \sqrt{\frac{-A_1 - \sqrt{A_1^2 - 4A_2}}{2\rho}} \quad \text{(S7)}$$

$$v_{\text{P-T}}(\alpha) = \sqrt{\frac{A_3}{\rho}}, \quad \text{(S8)}$$

where,

$$A_1 = -(\sin^2 \alpha C_{11} + \cos^2 \alpha C_{33} + C_{44}) \quad \text{(S9)}$$

$$A_2 = \sin^4 \alpha C_{11} C_{44} + \sin^2 \alpha \cos^2 \alpha (C_{11} C_{33} - C_{13}^2 - 2C_{13} C_{44}) + \cos^4 \alpha C_{33} C_{44}. \quad \text{(S10)}$$

$$A_3 = \sin^2 \alpha C_{66} + \cos^2 \alpha C_{44}. \quad \text{(S11)}$$

Through nonlinear χ^2 fitting of the BLS-measured sound velocities (i.e., *v* vs. *α*) with Equations (S6)-(S8), we obtained the elastic stiffness constants as well as their uncertainties.[12] The χ^2 is defined as

$$\chi^2 = \sum_i \frac{\left[v_{i,\,\text{fit}}(C_{11},\, C_{12},\, C_{13},\, C_{33},\, C_{44},\, \alpha) - v_{i,\,\text{exp}}(\alpha)\right]^2}{(\Delta v_{i,\,\text{exp}})^2}, \quad \text{(S12)}$$

where $v_{\text{i, fit}}$ and $v_{\text{i, exp}}$ are the fitted and experimental sound velocities, respectively, $\Delta v_{\text{i, exp}}$ is the uncertainty of the measured sound velocity, and the summation is over all experimental sound velocities. By considering the uncertainties of the measured angles, refractive indices, and phonon frequencies, we estimated $\Delta v_{\text{i, exp}}$ to be $0.02 v_{\text{i, exp}}$. We imposed the following constraints for the elastic stiffness constants[13]: (1) $C_{11} > |C_{12}|$, (2) $C_{44} > 0$, and (3) $C_{33}(C_{11} + C_{12}) > 2C_{13}^2$, which ensure positive Young's and shear moduli. The availability of experimental data for all the Q-L, Q-T, and P-T modes allows unique determination of C_{11}, C_{12}, C_{13}, C_{33}, and C_{44}.

After that, we calculated the engineering mechanical properties,[11] which include the in-plane Young's modulus ($E_{||}$), cross-plane Young's modulus ($E_{\perp}$), sliding shear modulus (G_{13}), torsional shear modulus (G_{12}), and two characteristic Poisson's ratios (ν_{31} and ν_{12}). Note that only five of the engineering mechanical properties are independent (typically, {$E_{||}$, $E_{\perp}$, G_{13}, G_{12}, ν_{31}} or {$E_{||}$, $E_{\perp}$, G_{13}, ν_{12}, ν_{31}} are chosen). Also note that to be consistent

with the direction-dependent thermal conductivity results, we used subscripts, "$_{\parallel}$" and "$_{\perp}$", to represent directions parallel and perpendicular to the sample film, respectively, rather than directions parallel and perpendicular to the "3"-axis (i.e., the symmetry axis), as seen in typical analysis of transversely isotropic materials. The relevant mechanical properties are summarized in Tables S4 and S5.

Table S4. Summary of elastic stiffness constants. Composition (Hec vol%, Hec wt%), density (ρ), refractive index (n), and elastic stiffness constants (C_{11}, C_{12}, C_{13}, C_{33}, C_{44}, and C_{66}) of the Hec/PVP hybrid Bragg stack films.

Sample ID	Hec vol%	Hec wt%	ρ (g/cm^3)	n	C_{11} (GPa)	C_{12} (GPa)	C_{13} (GPa)	C_{33} (GPa)	C_{44} (GPa)	C_{66} (GPa)
Hec0/PVP100	0	0	1.20	1.53*	10.4	5.2	5.2	10.4	2.6	2.6
Hec23/PVP77	23	40	1.55	1.54	67.6 ±1.7	28.0 ±1.9	6.0 ±1.2	13.2 ±0.3	1.5 ±0.1	19.8 ±0.5
Hec31/PVP69	31	50	1.62	1.50	84.3 ±1.5	34.0 ±1.8	6.1 ±1.5	15.7 ±0.3	1.2 ±0.2	25.1 ±0.5
Hec40/PVP60	40	60	1.74	1.47	101.6 ±1.8	35.9 ±2.2	6.5 ±1.8	19.7 ±0.4	1.4 ±0.1	32.9 ±0.6
Hec51/PVP49	51	70	1.97	1.45	134.1 ±2.2	55.9 ±2.9	7.7 ±2.3	23.0 ±0.5	1.1 ±0.1	39.1 ±0.9
Hec100/PVP0	100	100	2.70	1.40	242.5 ±6.2	103.2 ±6.7	40.9 ±5.2	35.0 ±0.9	1.0 ±0.1	69.6 ±1.2

*Source: https://refractiveindex.info/?shelf=organic&book=polyvinylpyrrolidone&page=Konig.

Table S5. Summary of engineering mechanical properties. Composition (Hec vol%, Hec wt%) and engineering mechanical properties of the Hec/PVP hybrid Bragg stack films. $E_{\parallel}$: in-plane Young's modulus; $E_{\perp}$: cross-plane Young's modulus; G_{13}, G_{23}: sliding shear moduli; G_{12}: torsional shear modulus; ν_{31}, ν_{32}, ν_{12}: Poisson's ratios (ν_{ij} represents the strain response in the j-direction due to a strain in the i-direction). Note that in Reference [11] ν_{31} was mistakenly denoted as ν_{13}.

Sample ID	Hec vol%	Hec wt%	$E_{\parallel}$ (GPa)	$E_{\perp}$ (GPa)	$\frac{E_{\parallel}}{E_{\perp}}$	G_{13} = G_{23} (GPa)	G_{12} (GPa)	$\nu_{31} = \nu_{32}$	ν_{12}
Hec0/PVP100	0	0	7.0	7.0	1	2.6	2.6	0.33 (assumed)	0.33 (assumed)
Hec23/PVP77	23	40	55.0 ±2.5	12.5 ±0.4	4.4	1.5 ±0.1	19.8 ±0.5	0.06 ±0.01	0.39 ±0.03
Hec31/PVP69	31	50	69.7 ±2.3	15.1 ±0.4	4.6	1.2 ±0.2	25.1 ±0.5	0.05 ±0.01	0.39 ±0.02
Hec40/PVP60	40	60	88.1 ±2.6	19.1 ±0.5	4.6	1.4 ±0.1	32.9 ±0.6	0.05 ±0.01	0.34 ±0.02
Hec51/PVP49	51	70	109.9 ±3.5	22.4 ±0.6	4.9	1.1 ±0.1	39.1 ±0.9	0.04 ±0.01	0.41 ±0.02
Hec100/PVP0	100	100	178.9 ±9.9	25.4 ±2.6	7.0	1.0 ±0.1	69.6 ±1.2	0.12 ±0.02	0.29 ±0.06

Section S4. Evaluation of interfacial thermal conductance

The series resistance model (SRM) shown in Figure 4F illustrates a hybrid stack unit cell consisting of one Hec layer and one PVP layer. We describe the corrugation of the Hec nanosheet (Figure 1B) by a thermal interface conductance, $G_{Hec/PVP}$. For the unit cell in Figure 4F, the total thermal resistance can be calculated as $\frac{d_{XRD}}{k_{\perp}} = \frac{d_{Hec}}{k_{Hec}} + \frac{d_{PVP}}{k_{PVP}} + \frac{2}{G_{Hec/PVP}}$, where d_{XRD} is the basal spacing as determined by XRD (Table S1), $k_{\perp}$ is the effective cross-plane thermal conductivity, d_{Hec} = 10 Å, and $d_{PVP} = d_{XRD} - d_{Hec}$. For the thermal conductivity of the two components, we used the following values: k_{Hec} = 5.71 W m^{-1} K^{-1}, and k_{PVP} = 0.17 W m^{-1} K^{-1}. The Hec/PVP interfacial conductance, $G_{Hec/PVP}$, is determined by least squares fitting of the experimental $k_{\perp}$ data (red solid line in Figure 4C) to be 89 ± 8 MW m^{-2} K^{-1}, which falls well into the range of interfacial conductance reported for inorganic/organic interfaces.[14] We also obtained the Hec/Hec interfacial conductance, $G_{Hec/Hec}$ = 219 ± 28 MW m^{-2} K^{-1}, by considering a unit cell consisting of one Hec layer and one Hec/Hec interface (i.e., $\frac{d_{XRD}}{k_{\perp}} = \frac{d_{Hec}}{k_{Hec}} + \frac{1}{G_{Hec/Hec}}$).

Section S5. Uncertainty analysis

The uncertainties (standard deviations) of the data reported in this work were analyzed by taking into account the instrument accuracy, repetitive measurements, and propagation of uncertainties. The sound velocities were estimated to have an error bar of 2%. The uncertainties of the elastic stiffness constants were determined according to Zgonik et al..[12] The uncertainties of the engineering mechanical properties were calculated according to principles of uncertainty propagation. For instance,

$$E_{\perp} = C_{33} - \frac{2C_{13}^2}{C_{11} + C_{12}} \tag{S13}$$

$$\Delta E_{\perp} = \sqrt{\sum_{ij=11,\,12,\,13,\,33} \left(\frac{\partial E_{\perp}}{\partial C_{ij}}\right)^2 \left(\Delta C_{ij}\right)^2 + \sum_{\substack{ij,\,kl=11,\,12,\,13,\,33 \\ ij<kl}} 2\frac{\partial E_{\perp}}{\partial C_{ij}}\frac{\partial E_{\perp}}{\partial C_{kl}}\left(\Delta C_{ij}\right)\left(\Delta C_{kl}\right)} \tag{S14}$$

where $\frac{\partial E_{\perp}}{\partial C_{11}} = \frac{2C_{13}^2}{(C_{11}+C_{12})^2}$, $\frac{\partial E_{\perp}}{\partial C_{12}} = \frac{2C_{13}^2}{(C_{11}+C_{12})^2}$, $\frac{\partial E_{\perp}}{\partial C_{13}} = -\frac{4C_{13}}{C_{11}+C_{12}}$, and $\frac{\partial E_{\perp}}{\partial C_{33}} = 1$. Similar expressions can be derived for the other engineering moduli and the Poisson's ratios.

The uncertainties of the thermal measurement results are analyzed in a similar way, i.e., by considering uncertainty propagation. For example,

$$k_{\|} = \alpha_{\|}\rho C_P \tag{S15}$$

$$\Delta k_{\|} = \sqrt{\left(\frac{\partial k_{\|}}{\partial \alpha_{\|}}\right)^2 \left(\Delta\alpha_{\|}\right)^2 + \left(\frac{\partial k_{\|}}{\partial \rho}\right)^2 \left(\Delta\rho\right)^2 + \left(\frac{\partial k_{\|}}{\partial C_P}\right)^2 \left(\Delta C_P\right)^2} \tag{S16}$$

where $\frac{\partial k_{\|}}{\partial \alpha_{\|}} = \rho C_P$, $\frac{\partial k_{\|}}{\partial \rho} = \alpha_{\|} C_P$, and $\frac{\partial k_{\|}}{\partial C_P} = \alpha_{\|}\rho$. Here we assumed negligible covariance terms. Similar expressions can be derived for $k_{\perp}$, $k_{\|}/k_{\perp}$, $\overline{\Lambda}$, etc.

The uncertainties of the data are reported as error bars in the figures shown in the main text and Supporting Information. For clarity, error bars smaller than the symbol size are not shown.

References

[1] a) J. Breu, W. Seidl, A. J. Stoll, K. G. Lange, T. U. Probst, *Chem. Mater.* **2001**, *13*, 4213-4220; b) M. Stöter, D. A. Kunz, M. Schmidt, D. Hirsemann, H. Kalo, B. Putz, J. Senker, J. Breu, *Langmuir* **2013**, *29*, 1280-1285.
[2] S. Rosenfeldt, M. Stöter, M. Schlenk, T. Martin, R. Q. Albuquerque, S. Förster, J. Breu, *Langmuir* **2016**, *32*, 10582-10588.
[3] D. M. Moore, R. C. Reynolds, D. M., *X-ray Diffraction and the Identification and Analysis of Clay Minerals*, Oxford University Press, Oxford, U.K., **1997**.
[4] E. S. Tsurko, P. Feicht, F. Nehm, K. Ament, S. Rosenfeldt, I. Pietsch, K. Roschmann, H. Kalo, J. Breu, *Macromolecules* **2017**, *50*, 4344-4350.
[5] a) A. Salazar, A. Mendioroz, R. Fuente, *Appl. Phys. Lett.* **2009**, *95*, 121905; b) A. Mendioroz, R. Fuente-Dacal, E. Apinaniz, A. Salazar, *Rev. Sci. Instrum.* **2009**, *80*, 074904.
[6] V. Singh, T. L. Bougher, A. Weathers, Y. Cai, K. Bi, M. T. Pettes, S. A. McMenamin, W. Lv, D. P. Resler, T. R. Gattuso, D. H. Altman, K. H. Sandhage, L. Shi, A. Henry, B. A. Cola, *Nat. Nanotechnol.* **2014**, *9*, 384-390.
[7] H. Hu, X. Wang, X. Xu, *J. Appl. Phys.* **1999**, *86*, 3953-3958.
[8] T. Still, *High frequency acoustics in colloid-based meso- and nanostructures by spontaneous Brillouin light scattering*, 1 ed., Springer-Verlag Berlin Heidelberg, **2010**.
[9] C. Klieber, *Ultrafast photo-acoustic spectroscopy of super-cooled liquids*, Massachusetts Institute of Technology, **2010**.
[10] a) S. P. Cheadle, R. J. Brown, D. C. Lawton, *Geophysics* **1991**, *56*, 1603-1613; b) M. Mah, D. R. Schmitt, *Geophysics* **2001**, *66*, 1217-1225.
[11] S. Cusack, A. Miller, *J. Mol. Biol.* **1979**, *135*, 39-51.
[12] M. Zgonik, P. Bernasconi, M. Duelli, R. Schlesser, P. Günter, M. H. Garrett, D. Rytz, Y. Zhu, X. Wu, *Phys. Rev. B* **1994**, *50*, 5941-5949.
[13] F. Mouhat, F.-X. Coudert, *Phys. Rev. B* **2014**, *90*, 4104.
[14] a) M. D. Losego, M. E. Grady, N. R. Sottos, D. G. Cahill, P. V. Braun, *Nat. Mater.* **2012**, *11*, 502-506; b) W. L. Ong, S. M. Rupich, D. V. Talapin, A. J. McGaughey, J. A. Malen, *Nat. Mater.* **2013**, *12*, 410-415.

Author Contributions

M.R., J.B., and G.F. initialized the idea. T.S. explored and optimized the Hec/PVP system, fabricated the hybrid Bragg stacks, and characterized their structures. K.R. conducted the BLS experiments. A.P. conducted and analyzed the lock-in thermography measurements to obtain the in-plane thermal conductivity. P.H. performed and analyzed the photoacoustic characterization to obtain the cross-plane thermal conductivity and measured the sample densities. A.F.K. conducted additional lock-in thermography measurements and contributed to the lock-in thermography analysis. A.L. conducted and analyzed the DSC measurements to obtain the specific heat. Z.W. analyzed the BLS data and established the correlation between the mechanical moduli and thermal conductivity. Z.W., M.R., J.B., and G.F. wrote the manuscript. All authors have given approval to the final version of the manuscript. †These authors contributed equally.
